# Anaemia and thrombocytopenia in patients with prostate cancer and bone metastases

**DOI:** 10.1186/1471-2407-10-284

**Published:** 2010-06-13

**Authors:** Carsten Nieder, Ellinor Haukland, Adam Pawinski, Astrid Dalhaug

**Affiliations:** 1 Department of Internal Medicine - Division of Oncology and Palliative Medicine, Nordland Hospital, Bodø, Norway; 2 Institute of Clinical Medicine, Faculty of Medicine, University of Tromsø, Tromsø, Norway

## Abstract

**Background:**

The purpose of this study was to determine the incidence, risk factors and prognostic impact of anaemia and thrombocytopenia in patients with bone metastases (BM) from prostate cancer.

**Methods:**

Retrospective cohort study including 51 consecutive patients treated at a community hospital. Twenty-nine patients (57%) received taxotere after diagnosis of BM.

**Results:**

Haemoglobin (Hb) ≤ 12.0 g/dL at BM detection was associated with shorter overall survival. During follow-up, 25 patients (49%) experienced episodes with Hb < 10 g/dL unrelated to side effects of cancer therapy. Fifteen patients required red blood cell transfusion. Median time from diagnosis of BM to Hb < 10 g/dL was 23 months. Median survival from Hb < 10 g/dL was 5.4 months. There was no factor predicting for Hb < 10 g/dL. Five patients (10%) developed thrombocyte (Trc) count <50 × 10^9^/L. All of these had previously received blood transfusion. Median interval from Hb < 10 g/dL to Trc < 50 × 10^9^/L was 2.5 months. Survival after thrombocytopenia was short (3 weeks to 4 months). Haematuria and subdural haematoma were among the causes of death.

**Conclusions:**

We found high rates of significant bone marrow failure in treatment-refractory patients. Both Hb < 10 g/dL and Trc < 50 × 10^9^/L predict for unfavourable survival.

## Background

Bone metastasis is a common complication in patients with advanced stage prostate cancer and might even be found already at first clinical diagnosis [1,2]. Depending on the extent of spread, bone marrow function might become compromised, resulting in anaemia und thrombocytopenia [3,4]. Prognosis after onset of anaemia und thrombocytopenia is not well described in the literature. In addition, factors predicting for these complications are poorly understood. To study the incidence, outcome and risk factors for anaemia and thrombocytopenia in men with prostate cancer and skeletal metastases, a retrospective cohort study was performed.

## Methods

A retrospective analysis, which included all patients with prostate cancer and bone metastases treated at the authors' institution during 2007 and 2008, was performed. The authors' institution is a community hospital in rural Norway, which is the only oncology care provider and services the complete population of the county, i.e. approximately 236,000 inhabitants. Thus, the 51 consecutive patients included in this study represent an unselected population. Follow-up information was available in all patients. Temporary anaemia, leuko- and thrombocytopenia episodes might result from chemotherapy or radioisotope toxicity, necessitating for example chemotherapy dose reduction. Such toxicity is reversible and not expected to predict short survival. The present analysis did not include reversible events in patients who received chemotherapy or radioisotopes at the time of the event. It is focused on anaemia and thrombocytopenia resulting from disease progression. The cut-off for low Hb was set at 10.0 g/dL as patients with higher values are not expected to receive red blood cell transfusion. Regarding Trc, 50 × 10^9^/L was chosen as higher values will not result in bleeding complications. The normal range for haemoglobin (Hb) was 13.4-17.0 g/dL. The normal range for thrombocytes (Trc) was 130-400 × 10^9^/L. We used the Kaplan-Meier method to generate actuarial survival curves. Patients without event were censored at last clinical follow-up. Survival was calculated from the date of imaging diagnosis of bone metastases (typically by isotope bone scan) or from development of Hb < 10 g/dL. Survival curves were compared with the log rank test. Wilcoxon- and Kruskal-Wallis-tests were used to compare the baseline characteristics between different groups. A p-value ≤ 0.05 was considered statistically significant.

### Results

The patient characteristics and length of follow-up are shown in Table 1. Treatment consisted of different types of androgen suppression regimens incl. steroids and palliative external beam radiotherapy in patients with bone pain, metastatic spinal cord compression or surgically stabilized pathological fractures. Administration of other treatments is also shown in Table 1. Twenty-nine patients (57%) received taxotere after diagnosis of bone metastases and 7 of these also proceeded to second-line treatment with mitoxantrone. The initial number of bone metastases on radioisotope bone scan was significantly higher in patients with synchronous presentation (25% with up to 10 foci, 55% with more than 10 foci and 20% with super scan) compared to metachronous presentation (52% with up to 10 foci, 45% with more than 10 foci and 3% with super scan), p = 0.05. Patients with synchronous presentation also had significantly higher median prostate-specific antigen (PSA) value, p < 0.01 (Table 1). No other significant differences in baseline characteristics were found between these two groups.

**Table 1:** Baseline characteristics of 51 men with prostate cancer and bone metastases

Parameter	All 51 patients	20 patients with bone metastases at first cancer diagnosis	31 patients with metachronous diagnosis of bone metastases
Median age, range (years)*	67, 56-86	64.5, 57-79	73, 56-86

Median age at first cancer diagnosis	66, 53-80	64.5, 57-79	68, 53-80

Median interval, range (months)	18, 0-159	0	46, 5-159

Median PSA, range (μg/L)*	51, 3.9-10,302	339, 42-10,302	21, 3.9-727

Median Hb, range (g/dL)*	13.6, 10.2-16.8	13.9, 10.9-16.8	13.4, 10.2-15.2

Median Trc, range (×10^9^/L)*	218, 137-447	295, 137-435	198, 143-447

Gleason score <7, 7, >7**	6, 9, 23 16%, 24%, 61%	2, 4, 9 13%, 27%, 60%	4, 5, 14 17%, 22%, 61%

Other distant metastases	17 33%	6 30%	11 35%

≤10 bone metastases, >10, superscan	21, 25, 5 41%, 49%, 10%	5, 11, 4 20%, 55%, 20%	16, 14, 1 52%, 45%, 3%

Initial prostatectomy or radical radiotherapy	8 16%	0	8 26%

Taxotere treatment	29 57%	12 60%	17 55%

Zoledronic acid treatment	41 80%	17 85%	24 77%

Radioisotope treatment	8 16%	5 25%	3 10%

Median follow-up of living patients, range (months)	26, 9-84	31, 12-84	22, 9-67

* when diagnosed with bone metastases

** unknown in 5 patients with synchronous and 8 patients with metachronous diagnosis

PSA: prostate-specific antigen, Hb: haemoglobin, Trc: thrombocytes

**Figure 1:**
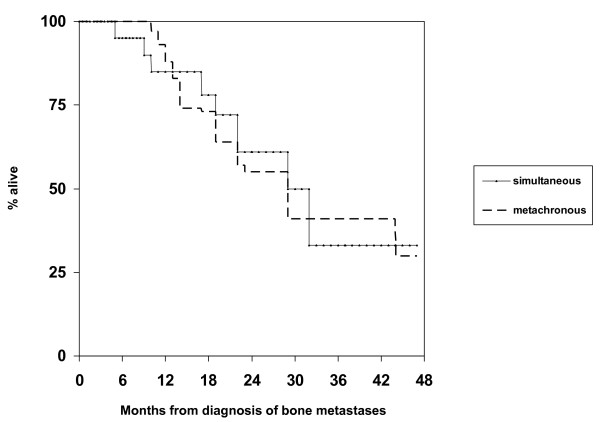
**Kaplan-Meier estimates of overall survival in 20 patients with bone metastases from prostate cancer, which were present at first cancer diagnosis, versus 31 patients who developed metachronous bone metastases during the course of disease, p = 0.6**.

All baseline characteristics shown in Table 1 were examined for their prognostic impact. Patients with bone metastases at initial diagnosis had a 2-year survival rate of 61% versus 55% in those with metachronous bone metastases (Figure 1, p = 0.6). PSA level significantly influenced survival, but only in patients with metachronous bone metastases. The 2-year survival rate was 30% in patients with PSA ≥ 21 μg/L at the time of bone metastases detection versus 71% in those with lower PSA, p < 0.01. While Hb at the time of bone metastases detection was not significant when using the median value as cut-off, a strong trend for correlation between Hb ≤ 12.0 g/dL and short survival was found, p = 0.03 (when correcting for the fact that 2 tests were performed, i.e. median Hb and Hb ≤ 12.0 g/dL, the Bonferroni correction requires p ≤ 0.025). Four of 5 patients with Hb ≤ 12.0 g/dL died within 18 months. None of the other factors significantly correlated with survival. Given these results, a multivariate analysis did not appear appropriate. Among the treatment-related factors, only the administration of chemotherapy significantly influenced survival. The 2-year rate was 68% in chemotherapy-treated patients versus 41% in others, p = 0.04.

**Figure 2:**
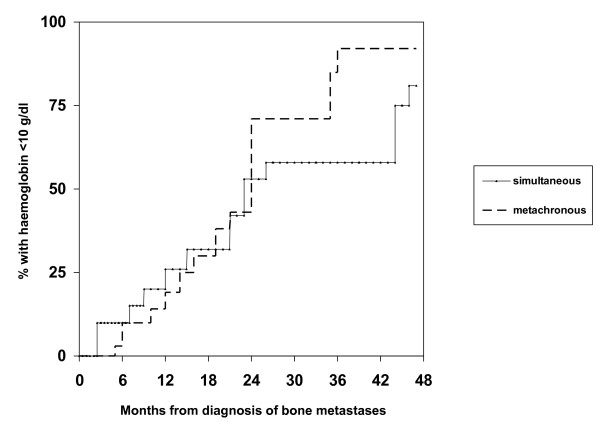
**Kaplan-Meier estimates of time to haemoglobin <10 g/dL in 20 patients with bone metastases from prostate cancer, which were present at first cancer diagnosis, versus 31 patients who developed metachronous bone metastases during the course of disease, p = 0.4**.

**Figure 3:**
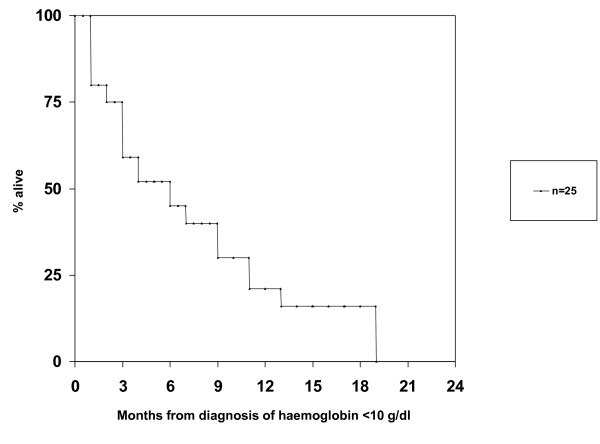
**Kaplan-Meier estimate of overall survival after detection of haemoglobin <10 g/dL in 25 patients**.

**Figure 4:**
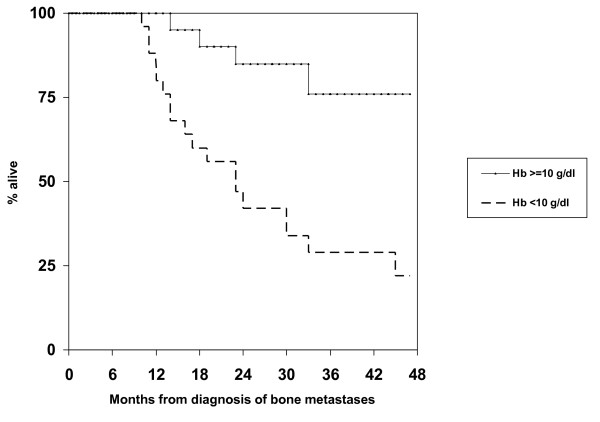
**Kaplan-Meier estimates of overall survival from first diagnosis of bone metastases in 25 patients who developed haemoglobin <10 g/dL during follow-up versus 26 patients who maintained higher haemoglobin levels, p = 0.01**.

Overall, 25 patients (49%) experienced episodes with Hb < 10 g/dL in the absence of chemotherapy and radioisotope injection, typically as a sign of disease progression indicating failure of the current treatment line. Fifteen of these patients (60%) required red blood cell transfusion (29% of all patients in the study). Erythropoiesis stimulating agents were not used. The median time from diagnosis of bone metastases to Hb < 10 g/dL was approximately 2 years (Figure 2). Median survival from Hb < 10 g/dL was 5.4 months (Figure 3). There was no factor predicting for episodes with Hb < 10 g/dL. Interestingly, patients having had Hb below median at diagnosis of bone metastases were not at increased risk of developing Hb < 10 g/dL during the course of disease. Their risk was 43% as compared to 59% in patients with Hb above median. Figure 4 shows that patients who maintained Hb ≥ 10 g/dL during follow-up had significantly longer survival from first diagnosis of bone metastases as compared to patients who developed Hb < 10 g/dL. Five patients (10%) developed episodes with Trc < 50 × 10^9^/L. All of these had previously experienced Hb < 10 g/dL and received red blood cell transfusion. Thus, 5 of 15 patients (33%) who had required transfusion also developed severe thrombocytopenia. The interval from Hb < 10 g/dL to Trc < 50 × 10^9^/L was 1-4 months, median 2.5 months. The outcome of these 5 patients is shown in Table 2. In spite of repeat platelet transfusion survival was short, ranging from 3 weeks to 4 months. No bleeding episodes were registered in patients who never presented with Trc < 50 × 10^9^/L. No patients developed severe leucocytopenia in the absence of chemotherapy administration or complications related to low white blood cell counts.

**Table 2:** Outcome in all 5 patients who developed thrombocyte (Trc) count < 50 × 10^9^/L after diagnosis of bone metastases

**Patient nr**.	Presentation	Minimum Trc count	Time from bone metastases to Trc < 50 × 10^9^/L	Previous systemic therapy	Outcome after Trc < 50 × 10^9^/L
1	Synchronous	15 × 10^9^/L	18 months	END, ZA, TAX, MITO	Died after 4 weeks, cause unknown

2	Synchronous	19 × 10^9^/L	16 months	END, ZA, TAX	Died from haematuria and kidney failure after 8 weeks

3	Synchronous	20 × 10^9^/L	27 months	END, ZA, TAX	Developed subdural haematoma but died from sepsis after 4 months

4	Synchronous	26 × 10^9^/L	4 months	END, ZA	Died from surgery complications (for pathol. fracture) after 3 weeks

5	Metachronous	30 × 10^9^/L	9 months	END, ZA	Died from subdural haematoma after 3 weeks

END: endocrine therapy, ZA: zoledronic acid, TAX: taxotere, MITO: mitoxantrone

## Discussion

The present study is to our best knowledge the only contemporary series examining the incidence, outcome and risk factors for development of anaemia and thrombocytopenia in patients treated for bone metastases from prostate cancer. To avoid confounding factors, reversible events caused by chemotherapy or radioisotope toxicity were not evaluated. Reversibility was determined by retrospective chart review. Beyond general limitations of retrospective studies, which might contain hidden sources of bias, one should be aware of the limited patient number and thus statistical power. We can not exclude the possibility that a larger study could have identified factors predicting for episodes with Hb < 10 g/dL. However, the data are derived from a representative unselected patient population, actually including all men with bone metastases from prostate cancer in a well defined geographical region. Therefore, it is likely that our findings apply to many men with bone metastases from prostate cancer treated outside of clinical trials by practicing oncologists. We had to arbitrarily define anaemia and thrombocytopenia. Other cut-off values might have been possible, but we decided to consider the probability for red blood cell transfusion and risk of bleeding when choosing Hb < 10 g/dL and Trc < 50 × 10^9^/L. All relevant clinical events were captured when applying these cut-off values. Geenen et al. have previously shown that the white blood cell system did not seem to be affected in patients with metastatic prostate cancer [5]. The present study confirms this result.

Treatment was individualised, taking into account age, organ function, performance status, symptoms etc. The majority of patients (57%) received taxotere after diagnosis of bone metastases and some patients also had second-line treatment with mitoxantrone. Administration of chemotherapy significantly influenced survival. The 2-year rate was 68% in chemotherapy-treated patients versus 41% in others. This difference is only partially attributable to treatment as this was a retrospective study where several sources of bias influenced the choice of treatment. Survival in most patients was 2-3 years, but 4 patients were alive more than 5 years after the detection of bone metastases.

It should also be noticed that androgen deprivation therapy might result in declining Hb, e.g., mean reduction by 1.1 g/dL in the study by Curtis et al. [6]. Beer et al. observed a mean decline of 0.54 g/dL 3 months after starting androgen deprivation therapy [7]. However, the mean level increased in patients with baseline level < 12 g/dL. A decline after 3 months was associated independently with shorter survival and progression-free survival. Already in a previous study, the same group had described an association between anaemia and shorter survival in men with newly diagnosed metastatic prostate cancer [8]. These recent results confirm established prognostic models such as the one developed by Halabi et al., which includes, e.g., Hb, alkaline phosphatase, lactate dehydrogenase and PSA [9]. Because the focus of the present study was on haematological events, detailed analyses of all prognostic factors for survival including alkaline phosphatase and lactate dehydrogenase were not performed. Other authors demonstrated that patients with lower Hb had more advanced disease on bone scan [10]. The time to development of bone metastases (synchronous versus metachronous presentation) and the number of foci on isotope bone scan had no influence on any outcome in the present study. The same holds true for age and distant metastases at non-skeletal sites. While Hb at the time of bone metastases detection was not significant when using the median value as cut-off, an association of Hb ≤ 12.0 g/dL and short survival might be present.

A large number of patients (49%) experienced episodes with Hb < 10 g/dL unexplained by chemotherapy and radioisotope toxicity, but reflecting disease progression. Sixty percent of patients with Hb < 10 g/dL required red blood cell transfusion (29% of all patients in the study). In a previous study, only 10% of patients became anaemic and 7.5% received red blood cell transfusion, but that study was limited to the final year of life and largely to the pre-taxotere era [4]. Notably the decision to transfuse and timing is somewhat subjective and varies from physician to physician. It should also be noted that we did not administer erythropoiesis stimulating agents, which might reduce the need for transfusion, given the debate around these agents and recent recommendations [11-13]. Median survival from Hb < 10 g/dL was 5.4 months. Thus, this factor predicts when the disease reaches a critical point where the remaining life time is very limited. No risk factors for development of Hb < 10 g/dL could be identified. Five patients (10%) developed episodes with Trc < 50 × 10^9^/L. All of these had previously experienced Hb < 10 g/dL and received red blood cell transfusion. Thus, 5 of 15 patients (33%) who had required transfusion also developed severe thrombocytopenia. Survival after detection of severe thrombocytopenia was short, ranging from 3 weeks to 4 months. Complications such as haematuria, subdural haematoma and the inability to recover from emergency surgery were among the causes of death.

## Conclusions

Declining bone marrow function continues to be a common event during the course of prostate cancer with skeletal metastases. It contributes significantly to morbidity and mortality and poses challenges to those involved in palliative care for these patients. The current survival figures after detection of Hb < 10 g/dL and Trc < 50 × 10^9^/L should be regarded as initial estimates, which need to be confirmed in larger studies.

## Competing interests

The authors declare that they have no competing interests.

## Authors' contributions

CN, EH and AD participated in the design of the study, EH, AD and AP collected patient data and follow-up information, CN carried out the statistical analysis, CN and AP drafted the manuscript. All authors read and approved the final manuscript.

## Pre-publication history

The pre-publication history for this paper can be accessed here:

http://www.biomedcentral.com/1471-2407/10/284/prepub
